# ECG Markers of Cardiovascular Toxicity in Adult and Pediatric Cancer Treatment

**DOI:** 10.1155/2021/6653971

**Published:** 2021-01-19

**Authors:** Ștefan Spînu, Gabriel Cismaru, Paul-Mihai Boarescu, Sabina Istratoaie, Alina Gabriela Negru, Cecilia Lazea, Simona Sorana Căinap, Daniela Iacob, Alin Ionut Grosu, George Saraci, Claudia Burz, Andrei Cosmin Cismaru

**Affiliations:** ^1^Oncology Institute “Prof. Dr. Ion Chiricuţă”, Cluj-Napoca, Romania; ^2^Doctoral School, “Iuliu Hațieganu” University of Medicine and Pharmacy, Cluj-Napoca, Romania; ^3^5th Department of Internal Medicine, Cardiology Rehabilitation, “Iuliu Hațieganu” University of Medicine and Pharmacy, Cluj-Napoca, Romania; ^4^Department of Pathophysiology, “Iuliu Hațieganu” University of Medicine and Pharmacy, Cluj-Napoca, Romania; ^5^Department of Pharmacology, Toxicology and Clinical Pharmacology, “Iuliu Hațieganu” University of Medicine and Pharmacy, Cluj-Napoca, Romania; ^6^“Victor Babeș” University of Medicine and Pharmacy, Timișoara, Romania; ^7^1st Pediatric Clinic, “Iuliu Hațieganu” University of Medicine and Pharmacy, Cluj-Napoca, Romania; ^8^2nd Pediatric Clinic, “Iuliu Hațieganu” University of Medicine and Pharmacy, Cluj-Napoca, Romania; ^9^3rd Pediatric Clinic, “Iuliu Hațieganu” University of Medicine and Pharmacy, Cluj-Napoca, Romania; ^10^Cardiology Department, Municipal Clinical Hospital, “Iuliu Hațieganu” University of Medicine and Pharmacy, Cluj-Napoca, Romania; ^11^“Iuliu Hațieganu” University of Medicine and Pharmacy, Cluj-Napoca, Romania; ^12^Department of Immunology and Allergology, “Iuliu Hațieganu” University of Medicine and Pharmacy, Cluj-Napoca, Romania; ^13^Research Center for Functional Genomics, Biomedicine and Translational Medicine, “Iuliu Hațieganu” University of Medicine and Pharmacy, Cluj-Napoca, Romania

## Abstract

When a cardiologist is asked to evaluate the cardiac toxic effects of chemotherapy, he/she can use several tools: ECG, echocardiography, coronary angiography, ventriculography, and cardiac MRI. Of all these, the fastest and easiest to use is the ECG, which can provide information on the occurrence of cardiac toxic effects and can show early signs of subclinical cardiac damage. These warning signs are the most desired to be recognized by the cardiologist, because the dose of chemotherapeutics can be adjusted so that the clinical side effects do not occur, or the therapy can be stopped in time, before irreversible side effects. This review addresses the problem of early detection of cardiotoxicity in adult and pediatric cancer treatment, by using simple ECG recordings.

## 1. Introduction

In the last twenty years, the survival and life expectancy of adults and children with cancer have risen significantly, mainly due to the new chemotherapy. However, chemotherapeutic agents have secondary and adverse effects, some of them dreadful. Their early recognition can prevent the development of associated sometimes fatal pathologies. Monitoring the cardiac side effects of chemotherapy is feasible generally using echocardiography, radionuclide ventriculography, dosing cardiac biomarkers [1] such as BNP and NT-proBNP [2], and ECG. Sometimes, these techniques may identify subclinical heart damage [3] before the clinical manifestation by heart failure, chronic coronary syndrome, or myocardial infarction. Therefore, an attempt was made to discover early markers of toxicity, and the purpose of this review is to present published data on ECG changes as markers of cardiac toxicity caused by chemotherapeutics. The 12-lead surface ECG is a simple examination that is performed quickly in about 3 minutes and can provide information on cardiotoxicity, which is mainly manifested by ischemic changes or by arrhythmias. Of course, there are more subtle changes, which can precede the installation of arrhythmias: for example, bifid and broad P wave lasting more than 120 ms that precedes the installation of atrial fibrillation or the prolonged QT interval > 500 ms that precedes in some cases the installation of torsade de pointes. Sometimes, the presence of multiple atrial ectopic beats may require stopping chemotherapy in order to prevent atrial fibrillation; the presence of numerous PVCs with multiple morphologies may require discontinuation of chemotherapy due to an increased risk of malignant ventricular arrhythmias such as polymorphic ventricular tachycardia or ventricular fibrillation. These ECG markers are easily recognizable by the clinical cardiologist or interventional arrhythmologist but are more challenging for an oncologist or general practitioner. The ECG does involve not only 12-lead recording but also derivatives such as recording with a monitor during hospitalization, single-lead or two-lead monitoring at home with a portable monitor (Omron, Heal Force Print 180 D, 180B), and monitoring by Apple devices, smartwatch, smartphones, Holter ECG/24 hours, exercise stress test, or electrophysiological study [4]. These are derivatives of the 12-lead ECG, and we will not refer to them in this review. The electrocardiographic changes given by chemotherapy can be transient, and therefore, other methods than the standard ECG are used to detect them. Generally, before starting chemotherapy, it is suitable for the patient to have a baseline ECG recording so that later, after starting the treatment, the measurements may be compared with the initial recording.

## 2. Arrhythmogenic Mechanisms of Chemotherapy

There are several mechanisms by which chemotherapy can become proarrhythmogenic (Table 1):

1. By the effect of direct damage to the myocardial cell with the release of natriuretic peptides BNP, NT-proBNP, and troponin, with the development of ischemic or nonischemic dilated cardiomyopathy, increased left ventricular filling pressures, and subsequently left atrial and fibrillation
2. Coronary spasm with the induction of myocardial ischemia or a direct effect of the chemotherapeutic on coronary vascularization with secondary ischemia, with or without myocardial necrosis and arrhythmogenesis by the formation of abnormal reentry circuits or abnormal depolarizations
3. Action at the level of ion channels with impaired ventricular depolarization or repolarization, prolongation of the QT interval, and induction of polymorphic ventricular tachycardia (torsade de pointes)
4. Direct action on the conduction system: sinus node, atrioventricular node, His, left or right branch, respectively, and Purkinje network

One of the most common side effects of chemotherapy that can be detected on the ECG in 12 leads is sinus bradycardia. Taxanes and angiogenesis inhibitors (thalidomide) can cause sinus bradycardia, most likely through a direct action on the sinus node. Nevertheless, taxanes can have effects at other levels of the atrioventricular conduction system. Thus, paclitaxel can affect the infrahisian conduction system (after the bifurcation of the His) and lead to the appearance of the right or left branch block. If the lesion is located at the suprahisan level, atrioventricular blocks of varying degrees, from 1 to 3, may occur. The conduction disorders generally occur within 4 hours of initiating the paclitaxel infusion and disappear after stopping the chemotherapeutic, usually within the first 48 hours [5]. The mechanism by which paclitaxel affects the conduction system is either directly by affecting the sinus node, atrioventricular node, and His-Purkinje system or indirectly by affecting the parasympathetic nervous system, which induces bradycardia or conduction disorders. Thalidomide-induced sinus bradycardia is also explained by the action on the sympathetic nervous system [6] and also the induction of a manifest clinical or subclinical hypothyroidism which is in turn associated with sinus bradycardia by intrinsic remodeling of the sinus node [7]. On the other hand, thalidomide has also been implicated in the development of rapid ventricular arrhythmias, especially ventricular tachycardia [8].

Another mechanism promoting cardiac arrhythmias is through myocardial ischemia [9] or even necrosis if myocardial ischemia persists for a long time. Thus, alkylating agents cisplatin, cyclophosphamide [10], ifosfamide, and melphalan may promote coronary vasospasm, cardiomyocyte damage, and endothelial damage. Up to 10% of cisplatin users develop atrial and ventricular arrhythmias within the first 24 hours-3 days of initiating treatment, with the disappearance of these side effects within approximately 1 week. Melphalan has also been implicated in the genesis of episodes of atrial fibrillation or atrial flutter [11].

Anthracyclines cause atrial and ventricular arrhythmias by inducing structural cardiomyopathy associated with decreased left ventricular systolic function with altered ejection fraction. This decrease in ejection fraction leads to increased left ventricular end-diastolic pressure and increased left intra-atrial pressure and favours atrial arrhythmias such as extrasystoles, atrial tachycardia, or atrial fibrillation. On the other hand, the marked decrease of the ejection fraction may favour ventricular arrhythmias: premature ventricular contractions, ventricular tachycardia, and ventricular fibrillation.

5-Fluorouracil validates its arrhythmogenic effects through coronary vasospasm and the myocardial ischemia it induces. Like 5-FU, interleukin-2 promotes arrhythmias by inducing vasospasm with consequent prolonged myocardial ischemia or myocardial inflammation (myocarditis) [12].

## 3. ECG Modifications and Arrhythmias Induced by Different Chemotherapeutics

### 3.1. Anthracyclines

Anthracyclines are currently used to treat leukemias, lymphomas, breast cancer, and solid pediatric tumors. Generation I anthracyclines (daunorubicin and doxorubicin) have as a side effect the irreversible development of nonischemic dilated cardiomyopathy, and their effects are cumulated with increasing doses and duration of use (an incidence of 5-8% is observed at a cumulative dose of 450 mg/m^2^) [13].

A study by Kilickap's team that involved Holter EKG monitoring for 48 hours of patients immediately after doxorubicin infusion showed a paroxysmal atrial fibrillation rate of 10.3% [14]. However, when ECG monitoring was performed at each visit for the continuation of chemotherapy, a 6% incidence of this arrhythmia was recorded [15]. The highest detection rate (56.6%) was objectified by interrogating the implantable defibrillator in patients with cardiac dysfunction associated with chemotherapy [16]. The same study shows that the incidence of nonsustained ventricular tachycardia can reach up to 73.9% of cases, similar to those in the control group, of patients with nonischemic cardiomyopathy, not related to anthracyclines (was not significantly different from non-anthracycline-related cardiomyopathy and dilated cardiomyopathy or ischemic heart disease) [16]. However, premature ventricular contractions remain the most common form of anthracycline-induced ventricular arrhythmia (the number of bigeminal ventricular extrasystoles increased significantly, *p* < 0.05) [17].

A study analyzing the effects of epirubicin on QTc interval dispersion (defined as the difference between the maximum and minimum QT intervals on the recorded electrocardiogram) showed an increase in this parameter in all patients included in the research. When dexrazoxane was administered in addition to epirubicin, the dispersion of the QT interval decreased statistically (*p* < 0.05) compared to the group without dexrazoxane [18].

### 3.2. Alkylating Antineoplastic Agents

Alkylating agents are frequently used before stem or bone marrow transplantation. Several factors increase the risk of developing this melphalan-induced arrhythmia, including advanced age (over 63 years: risk ratio: 4.8 (95% confidence interval (CI) 3.2-7.6), *p* < 0.001), dilated left atrium over 33 cc/m^2^ (risk ratio: 2 (95% CI 1.3-3.1), *p* < 0.001), left ventricular systolic dysfunction (*p* < 0.001), and even cardiac amyloidosis (this was not significant, *p* = 0.08) [19, 20]. Besides, when a supraventricular arrhythmia occurs after hematopoietic stem cell transplantation, the prognosis of these patients worsens as observed in a study conducted by Tonorezos et al. on a group of 1177 patients. Of these, those with atrial fibrillation or flutter had a higher risk of in-hospital death (28% vs. 3%, *p* < 0.001) and also one year after the intervention (41% vs. 15%; *p* < 0.001). Practically, the existence of arrhythmias in post-stem cell transplant patients has been an independent predictor of mortality with a greater risk for death within a year of transplant (odds ratio 3.5 (95% CI 2.1-5.9; *p* < 0.001)) [21].

Busulfan is another chemotherapeutic belonging to the alkylating agent class. The incidence of developing atrial fibrillation was up to 6.4% when used in combination with cyclophosphamide [22].

In conclusion, during treatment with alkylating agents, patients should be monitored for atrial fibrillation with melphalan and busulfan and also for the development of structural abnormalities that may be responsible for ventricular arrhythmias with cyclophosphamide and ifosfamide.

### 3.3. Anti-HER2 Agents

An analysis that included over 8000 patients treated with trastuzumab reported an incidence of atrial fibrillation of 1.2% after trastuzumab use (95% CI 0.56-2.68) [23]. On the other hand, a study published in 2015 by Pivot and colleagues compared the cardiac toxicity generated by trastuzumab after 6 months and 12 months of adjuvant treatment, respectively. Of the 3380 enrolled patients, only 0.65% had NYHA III/IV heart failure classes in the one-year treatment arm and 0.53% in the six-month treatment arm, with no statistically significant difference between the two groups. For NYHA I/II heart failure classes, the number of cases was significantly higher in the one-year treatment group (5.9%) than in the group receiving 6 months of treatment (3.4%) [24]. When trastuzumab was used after a “dose-dense” (accelerated) regimen of anthracyclines and taxanes, the enrolled patients showed a significant decrease in LVEF in a tiny percentage (1%) [25].

The use of trastuzumab with paclitaxel after an anthracycline and cyclophosphamide induction regimen resulted in symptomatic heart failure in 4% of patients enrolled in the study (95% CI 0.5-13.2), and in 21% of them, a decrease in LVEF below 50% was noticed (95% CI 11.1-34.7) [26].

Another study that evaluated the safety of administration of trastuzumab in the elderly found that of the 22 patients, only 2 had a 10% asymptomatic decrease in LVEF [27].

More extensive studies of approximately 45,000 women with a mean age of 76.2 years showed that the 3-year incidence of cardiomyopathy or heart failure of any grade was 32.1% in the trastuzumab-only group and 41.9% in patients who also received anthracyclines compared with no adjuvant therapy (18.1%, *p* < 0.001) [28].

Trastuzumab-emtansine (T-DM1), a combination used in the second line of treatment after trastuzumab, did not cause any significant cardiovascular events (including symptomatic heart failure) in the 153 patients evaluated in Krop et al.'s study [29].

Lapatinib is a safer product than trastuzumab in that it rarely induces left ventricular dysfunction and arrhythmias [30].

### 3.4. Tyrosine Kinase Inhibitors

These products do not induce structural abnormalities of the myocardium but may prolong the QT interval and induce ventricular arrhythmias such as torsade de pointes.

Ibrutinib is a Bruton tyrosine kinase inhibitor used in B-line haematological malignancies such as chronic lymphocytic leukemia and mantle cell lymphoma. A study conducted by Yun et al., published in 2017, shows an incidence of atrial fibrillation/flutter of 8.18% in patients treated with ibrutinib compared to placebo (8.18% vs. 0.93%, RR = 8.81, 95% CI 2.70-28.75, *p* < 0.001). It should be remembered that the risk of arrhythmia is proportional to the dose and time of treatment [31]. According to the HELIOS phase 3 trial published in 2018 in the *Leukemia* journal, the rate of occurrence of atrial fibrillation/flutter as an adverse event was reported at 4.9% [32]. Another study, published in the *NEJM*, showed similar incidences of atrial fibrillation of 6%. In 25% of the patients, it was necessary to stop the treatment, but for the rest, no intervention was needed [33]. Although ibrutinib carries a relatively high risk of atrial fibrillation, it also acts as an antiplatelet agent, associated with an increased risk of bleeding. Therefore, the use of antivitamin K in this category of patients has been discouraged. It seems that the new oral anticoagulants such as dabigatran, rivaroxaban, or apixaban have a higher safety profile [34].

Regarding Bruton tyrosine kinase inhibitors inducing ventricular arrhythmias, there is a reported incidence of 678 events/100,000 patients. Surprisingly, ibrutinib does not prolong the QTc interval; it even shortens it, although it does induce a risk of ventricular tachycardia [35].

### 3.5. Antimicrotubule Agents

In a study conducted by Rowinsky et al., 2 of the 140 patients treated with paclitaxel had a high-grade atrioventricular block and therefore required the implantation of a pacemaker. However, EKG monitoring of patients during injection is not indicated [36].

### 3.6. Immunomodulating Agents

Thalidomide is an agent used in the treatment of multiple myeloma that can cause bradyarrhythmias, including different types of atrioventricular block, both alone and in combination with other chemotherapeutics. Sinus bradycardia has been reported in 26% up to 53% of patients and most often resolves within 12-21 days of discontinuation of treatment [7].

Thalidomide was also associated with atrial fibrillation, with an incidence of 4.7% versus 3.4% in the placebo-treated arm. Therefore, cardiac monitoring is recommended in all patients treated with this immunomodulator [37].

Lenalidomide is another immunomodulator used in both multiple myeloma and myelodysplastic syndrome. It can induce supraventricular arrhythmias with an incidence ranging from 4.6 to 7% when used in combination with dexamethasone [38].

### 3.7. Amsacrine

This substance is used for the treatment of acute myeloid leukemia and electrophysiologically acts similar to anthracyclines. It can cause atrial and ventricular arrhythmias, respectively, and QT prolongation. However, proarrhythmic effects are rare and were reported in 0.7% in a study of 5340 patients [39]. The administration of amsacrine is prone to hydroelectrolytic disturbances which may eventually lead to arrhythmias. Therefore, the use of this chemotherapeutic requires strict monitoring of the electrolytes (especially the level of potassium) to be administered safely, even in patients with left ventricular dysfunction [40].

### 3.8. Interleukin 2 (IL-2)

The mechanism by which IL-2 induces cardiac arrhythmias is the increase of capillary permeability with tissue extravasation and hypotension and tachycardia. If those modifications occur in a structurally normal heart, they are not arrhythmogenic, but when they occur in an ischemic heart, they can produce atrial and ventricular arrhythmias. Another speculated mechanism is the action that different vasopressors have on the electrical system of the heart. Ventricular arrhythmias are also possible, but the frequency of life-threatening ventricular tachycardias is low, between 0.4 and 1.1% [41].

### 3.9. Trisenox (Arsenic Trioxide)

Arsenic trioxide inhibits fast and slow potassium channels and activates ATP-dependent potassium channels. This is the electrophysiological mechanism underlying QT prolongation with a high risk of torsade de pointes. Approximately 38% of patients who are treated with arsenic trioxide develop QT prolongation > 450 ms and 27% > 500 ms [42]. The degree of QT prolongation was higher in male patients during the first cycle of treatment and also in patients with hypokalemia regardless of gender. Corrected QT intervals in these patients normalized up to the second cycle of chemotherapy, so it was considered that arsenic trioxide did not cause a permanent prolongation of the QTc interval [42]. Because life-threatening arrhythmias are rarely associated with arsenic trioxide, caution is advised in the use of QTc with the Bazett formula since the risk of cardiac toxicity may sometimes be overestimated, and therefore, cancer treatment may be unnecessarily discontinued. In these situations, alternative correction formulas are recommended [43]. Fortunately, the doses used in practice at this time are optimized so that ventricular arrhythmias occur with a fair frequency, but it is still necessary to monitor electrolytes and ECG in those patients, both before and during treatment [44].

### 3.10. Histone Deacetylase Inhibitors

These products, which are used to treat cutaneous T-cell lymphoma and multiple myeloma, can prolong the QT interval, and there have been reported cases of sudden death after starting treatment. For these reasons, they are contraindicated in the case of a QT interval over 450 ms. The underlying mechanism of arrhythmogenesis is not fully understood. However, it is currently accepted that these inhibitors interact with potassium hERG channels [45].

### 3.11. Antimetabolites

The most common side effects with 5-fluorouracil and capecitabine are chest pain, with or without EKG signs of ischemia. The mechanism of action is a coronary spasm. A spasm was demonstrated by reproduction in the radial artery after administration of 5-fluorouracil. The use of calcium channel blockers and long-acting nitrates has been proposed to counteract the vasospastic effects of antimetabolites [46].

## 4. Arrhythmias Determined by Long QT Interval

**Figure 1:**
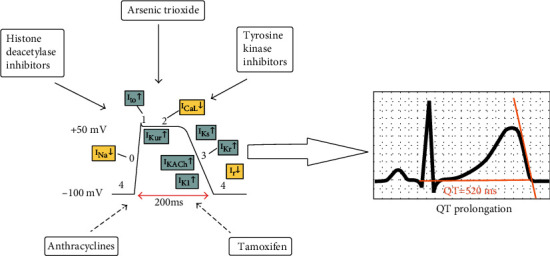
Chemotherapy that induces QT prolongation. Different agents act on different or more ionic channels prolonging ventricular depolarization and depolarization. A QT prolongation of >500 ms is considered dangerous and should lead to treatment cease.

One of the most straightforward cardiotoxicity markers that can be measured on the surface ECG is the QT interval (Figure 1), an easy to measure, standardized interval, the most used method being the tangent method in derivation II or V5. Because ECG paper is often marked with lines or squares delimiting 40 ms, the measurement is easy considering the number of squares found along the length of the QRS complex and the T-wave. This interval represents depolarization along with ventricular repolarization. The QT interval measurement must not include the U-wave unless there is an evident fusion between T and U.

This QT interval is vital in arrhythmogenesis because when prolonged, it can be associated with polymorphic ventricular tachycardia, in this case called torsade de pointes. Not all polymorphic tachycardia is torsade de pointes but only that which is accompanied by an extended QT interval. In other cases, the term “torsade-like” can be used.

Due to the fact that the QT interval can be longer in the case of bradycardia and shorter in the case of tachycardia, there are mathematical formulas for correcting the QT interval depending on the frequency: Bazett, Fridericia, Framingham, and Hodges. It is considered that a corrected QT interval < 450 ms in men and <460 ms in women is normal. When the QT interval is >500 ms, the risk for torsade de pointes is high. Intervals between 450 and 500 ms considered the “grey area” should be monitored by serial recordings, and serum electrolytes should be checked for hypokalemia or hypomagnesaemia.

The 450 ms limit of the QT interval is considered too restrictive in cancer patients because if this limit was to apply, then over 10% of patients receiving chemotherapy would have to give up perhaps life-saving therapy [47]. On the other hand, in oncology patients, there have been found variations of the QT interval up to 60 ms within 24 hours. Thus, in cancer patients, a prolonged QT interval > 480 ms or an increase of >100 ms after the initiation of chemotherapy is considered significant [48, 49].

Cancer patients who are treated with antiarrhythmic medication for heart disease are at risk of developing drug interactions with the possibility of prolonging the QT interval. Class IA (quinidine and procainamide) and class III antiarrhythmics (amiodarone and sotalol) prolong the QT interval by their particular mechanism of action on ion channels. On the other hand, antiemetic drugs such as ondansetron and droperidol also prolong the QT interval. Among analgesics, methadone, an opioid derivative, also prolongs the QT interval (+14.1 msec, *p* < 0.001) [50]. Commonly used antineoplastic medication may also have the effect of prolonging the QT interval: tyrosine kinase inhibitors (rituximab, dasatinib, lapatinib, nilotinib, and sorafenib), anthracyclines (doxorubicin and daunorubicin), and antimetabolites (capecitabine, panobinostat, romidepsin, and vorinostat). These associations with antiarrhythmics should be avoided to prevent malignant ventricular arrhythmias such as torsade de pointes [51].

Arsenic trioxide is known to prolong the QT interval, which is why therapy should be stopped if the interval is prolonged >500 ms. When the interval decreases to 460 ms, the treatment can be resumed. Shen et al. [52], Niu et al. [53], and Unnikrishnan et al. [54] have published case reports of torsade de pointes in patients being treated with arsenic trioxide. However, there is also stronger evidence than isolated published cases. Thus, Ohnishi et al. [55] reported 8 cases of QT prolongation after arsenic trioxide infusion, but none of the 8 patients showed torsade de pointes. The most extensive patient studies of arsenic trioxide included approximately 100 patients. Thus, the study of Barbey et al. [42] on 99 patients observed QT prolongation over 500 ms in 26% of individuals. Only one of these patients had torsade de pointes, but it was also associated with hypokalemia. Also, Roboz et al. [43] in a group of 113 patients observed QT prolongation > 500 ms in 12% of individuals, and none of them showed torsade de pointes.

### 4.1. Tyrosine Kinase Inhibitors

Nilotinib was associated with 5 to 15 ms QT prolongation, but this prolongation was not associated with polymorphic ventricular tachycardia [56]. On the other hand, in the study of Tam et al. [57], nilotinib administered to healthy volunteers resulted in an average QT prolongation of 18 ms. On subgroup analysis, 1.9% and even 2.5% of patients with chronic myeloid leukemia presented QT prolongation. Of all patients treated with nilotinib from Tam et al.'s study, 0.3% died suddenly, and it was assumed that QT interval prolongation had an involvement, although no direct relationship between had been demonstrated. Lu et al. [58]. have also shown that dasatinib, sunitinib, and nilotinib can prolong the QT interval. Studies with Vandetanib have shown a QT prolongation in 9% to 61% of the patients [59]. In Wells et al.'s [60] and Natale et al.'s [61] studies, QT prolongation occurred in approximately 5.1% of patients; only one of the patients presented torsade de pointes; in all other patients, the QT prolongation had no arrhythmic consequence. In these 2 studies that we have mentioned, the definition of the prolonged QT interval was >550 ms or an increase of >100 ms between 2 consecutive measurements. Zang et al. [62]. performed a systematic review and meta-analysis of Vandetanib' studies related to QT prolongation. They showed that QT prolongation > 450/460 ms occurred in 16.4% (95% CI 8.1–30.4) of patients and >500 ms prolongation occurred in 3.7% of patients (95% CI 1.7–7.8).

### 4.2. Histone Deacetylation Inhibitors

Vorinostat has been incriminated in the prolongation of the QT interval complicated with polymorphic ventricular tachycardia in a case report published by Lynch et al. [63]. It is important to mention that the patient associated hypokalemia. Probably hypokalemia, more than chemotherapy, was involved in the development of malignant ventricular arrhythmias, and this patient would have had a congenital long QT syndrome that could have been exposed by vorinostat. However, no genetic study has been done to confirm this hypothesis. Romidepsin has been incriminated in several cases of sudden cardiac death, but there has been no clear relationship between QT prolongation and death, as patients did not have an ECG recording before death [64, 65]. In the study of Piekarz et al. [64], QT prolongation was approximately 15 ms after administration of romidepsin. This prolongation is insignificant and does not justify stopping chemotherapy that might be life-saving. Last but not least, panobinostat is another histone deacetylation inhibitor, which has also been shown to prolong the QT interval up to 20 ms [65]. In conclusion, histone deacetylation inhibitors may prolong the QT interval, but no clear association with arrhythmic events such as torsade de pointes has been demonstrated.

### 4.3. Anthracyclines

Even though the most common side effect of anthracyclines is structural impairment of the left ventricle with decreased LV ejection fraction, to a lesser extent, anthracyclines may alter the QT interval. In the study of Galetta et al. [18], epirubicin produced variable QT prolongation when administered to patients with non-Hodgkin's lymphoma (all the patients showed increased QT dispersion (44.3 ± 8.4 vs. 68.4 ± 11.4 ms, *p* < 0.001) and QTc dispersion (46.2 ± 6.2 vs. 72.42 ± 8.4 ms, *p* < 0.001) after epirubicin-based chemotherapy in non-Hodgkin lymphoma patients). Also, in the study of Nousiainen et al. [66], QTc dispersion increased from 26.5 ± 2.5 to 39.0 ± 3.5 ms (*p* = 0.039). Five patients (18%) developed QT dispersion exceeding 50 ms. At the same time, Liu et al. [67] showed in experimental studies on rabbit myocytes that tamoxifen can prolong the QT interval.

## 5. ECG Changes Produced by Chemotherapy in Children

As in adults, chemotherapy can have cardiac toxicity in children. But children have 2 particularities: First, their heart is constantly developing, and the structure that has been affected by chemotherapy will increase with the growth of the child's heart, so in the following years, the injured structure will become larger [68]. Second, the survival of children is generally higher than that of adults; therefore, on the one hand, it is important to limit the cardiac toxic effect of chemotherapy which will last for years; on the other hand, if the toxic effect occurred, the evolution of left ventricular dysfunction should be blocked to prevent the development of clinical manifest heart failure. As for arrhythmias, they generally appear in the acute phase and disappear after stopping the chemotherapeutic. If arrhythmias occur in the chronic, postadministration phase, then antiarrhythmic drugs are generally needed to control arrhythmias.

Up to 25% of children who are treated with anthracyclines may have electrocardiogram changes [69]. These can be QT interval prolongation, ischemic or nonischemic T-wave and ST segment changes, bundle branch blocks or atrioventricular blocks, decreased QRS complex amplitude, and electric axis change. In addition, atrial or ventricular arrhythmias with different severity ranging from premature contractions to tachycardia were described. In the study of Larsen et al. [70] performed on 100 children with an average age of 15 years, 73 treated with anthracyclines and 27 treated with anthracyclines plus radiotherapy, minor arrhythmias were detected, such as rare atrial or ventricular premature contractions, as well as major arrhythmias. Among these are sustained supraventricular tachycardia or ventricular tachycardia that occurred especially at high doses of anthracyclines > 200 mg/m^2^. Furthermore, prolonged QT interval > 480 ms was found in approximately 14% of children. Another study by Steinherz and Steinherz [71] on 100 children identified ECG changes in 13 of them after anthracyclines. One of the 100 died suddenly, without any ECG changes, and 2 died due to arrhythmias. In another study, Lipshultz et al. [72] found 5% nonsustained ventricular tachycardia in children treated with doxorubicin. Amsacrine [73] can also cause ECG changes in children and usually occur within the first minutes or hours of administration. These are QT prolongations, ST and T-wave ischemic changes or nonspecific nonischemic changes, and atrial or ventricular tachyarrhythmias. Usually, these changes occur from the first dose and can be quickly detected by ECG. If the patient has an underlying hypokalemia, then the depolarization and repolarization changes given by amsacrine may be exacerbated by hypokalemia, so serum potassium levels should be monitored during therapy.

Massin et al. [74] studied severe arrhythmias that occur in the first 24 hours in 33 children with various tumors treated with chemotherapy. Two patients developed sinoatrial block or atrioventricular block during the first 4 hours of daunorubicin infusion, 8 children had atrial and ventricular premature beats or bursts of premature beats during the combination of vincristine+daunorubicin or vincristine+cyclophosphamide, and none of the children presented life-threatening arrhythmias.

In a study by Mulrooney et al. [75], 2715 children who survived neoplastic disease have been checked for ECG changes, which were interpreted as chronic side effects due to chemotherapy. Thus, 99 individuals were identified with pathological Q-waves as signs of old myocardial infarction, 5 with left branch block, 13 with right branch block, 4 with bifascicular block, 8 with significant QT prolongation, and none with atrial flutter or atrial fibrillation. In total, major ECG changes were present in 290 of 2715 patients (approximately 10%) and minor ECG changes in 565 (23.3%). Minor changes included atrial or ventricular premature beats, nonspecific T-wave or ST segment changes, low QRS, and deviation of the heart's electrical axis.

Newer studies are trying to determine whether lower chemotherapy doses that do not induce ECG changes still remain effective for the suppression of neoplastic disease. Researchers are trying to verify if lower doses that do not cause an excessive increase in QT interval > 480 ms or that produce only benign atrial or ventricular premature beats are still effective in controlling the child's neoplastic disease. The results of the studies would further benefit children, as toxic effects might affect a developing, immature heart.

## 6. Conclusions

Oncological treatment requires a good collaboration between the oncologist and the cardiologist. Even if new drugs increase the life expectancy of cancer patients, death may be due to a therapeutic dosing error, due to proarrhythmic side effects, or due to impaired left ventricular ejection fraction. For these reasons, some oncology clinics have hired a cardiologist who can monitor the evolution of heart function during chemotherapy by ECG, Holter ECG, and echocardiography. The cardiologist must know the limits that are acceptable for subclinical cardiac toxicity such as a mild but reasonably prolonged QT interval below 480 ms, the presence of benign arrhythmias such as atrial or ventricular premature beats, the presence of insignificant ST segment and T-wave changes, and changes in the heart axis. All of these ECG insignificant changes should not stop the child or adult from receiving a potentially life-saving therapy. When the oncology-cardiologist is not available, close collaboration with a cardiology clinic or outpatient cardiac clinic with experienced physicians in monitoring toxic effects of chemotherapy is required.

## Figures and Tables

**Table 1:** Proarrhythmic risk of chemotherapy: atrial, ventricular, and QT prolongation.

	Atrial arrhythmias 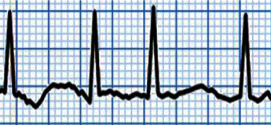	Ventricular arrhythmias 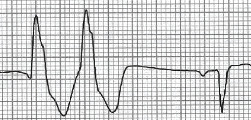	QT prolongation 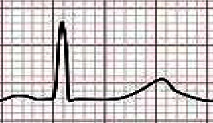
Anthracyclines	Atrial fibrillation with doxorubicin	Small case series	Rare cases
Antimetabolites	Small case series	Small case series	Rare cases
Cyclophosphamide	Rare cases	Never	Never
Melphalan	Atrial fibrillation and flutter	Never	Never
Trastuzumab	Rare cases	Rare cases	Never
Tyrosine kinase inhibitors	Atrial fibrillation with ibrutinib	Large studies: PVCs and VT	Small studies QT prolongation of 5-15 ms No QT prolongation with ibrutinib
Antimicrotubule agents	Rare cases	Small case series	Never
Arsenic trioxide	Rare cases	Small studies	Small studies showed QT prolongation > 450 ms and even >450 ms
Thalidomide	Atrial fibrillation	Small case series	Never
Histone deacetylase inhibitors	Never	Large studies: PVCs and VT	Small studies Therefore contraindicated if QT > 450 ms
IL-2	Small studies	Small studies	Never
Amsacrine	Small studies	Small case series	Rare cases, associated with hypokalemia

Large studies demonstrated atrial arrhythmias for melphalan, ventricular arrhythmias for tyrosine kinase inhibitors and histone deacetylase inhibitors, and QT prolongation for tyrosine kinase inhibitors, histone deacetylase inhibitors, and arsenic trioxide.
